# The neural correlates of regulating another person's emotions: an exploratory fMRI study

**DOI:** 10.3389/fnhum.2014.00376

**Published:** 2014-06-06

**Authors:** Glyn P. Hallam, Thomas L. Webb, Paschal Sheeran, Eleanor Miles, Karen Niven, Iain D. Wilkinson, Michael D. Hunter, Peter W. R. Woodruff, Peter Totterdell, Tom F. D. Farrow

**Affiliations:** ^1^Sheffield Cognition and Neuroimaging Laboratory, Academic Clinical Psychiatry, Department of Neuroscience, University of SheffieldSheffield, UK; ^2^Department of Psychology, University of YorkYork, UK; ^3^Department of Psychology, University of SheffieldSheffield, UK; ^4^Department of Psychology, The University of North Carolina at Chapel HillChapel Hill, NC, USA; ^5^School of Psychology, University of SussexBrighton, UK; ^6^Manchester Business School, University of ManchesterManchester, UK; ^7^Academic Unit of Radiology, University of SheffieldSheffield, UK

**Keywords:** fMRI, emotion regulation, interpersonal emotion regulation, empathy, social cognition

## Abstract

Studies investigating the neurophysiological basis of *intra*personal emotion regulation (control of one's own emotional experience) report that the frontal cortex exerts a modulatory effect on limbic structures such as the amygdala and insula. However, no imaging study to date has examined the neurophysiological processes involved in *interp*ersonal emotion regulation, where the goal is explicitly to regulate another person's emotion. Twenty healthy participants (10 males) underwent fMRI while regulating their own or another person's emotions. Intrapersonal and interpersonal emotion regulation tasks recruited an overlapping network of brain regions including bilateral lateral frontal cortex, pre-supplementary motor area, and left temporo-parietal junction. Activations unique to the interpersonal condition suggest that both affective (emotional simulation) and cognitive (mentalizing) aspects of empathy may be involved in the process of interpersonal emotion regulation. These findings provide an initial insight into the neural correlates of regulating another person's emotions and may be relevant to understanding mental health issues that involve problems with social interaction.

## Introduction

The adaptive control of emotional experience plays a critical role in daily functioning and mental health (Gross and John, 2003). The regulation of one's own emotions is termed “intrapersonal emotion regulation” (sometimes referred to as “intrinsic” regulation; Zaki and Williams, 2013), whereas regulation directed toward another person's emotions is termed “interpersonal emotion regulation” (Niven et al., 2009, also known as extrinsic emotion regulation; Zaki and Williams, 2013). A number of different strategies for interpersonal emotion regulation have been identified. For example, if a friend is sad then one might try to help them think about the situation differently in an effort to alleviate the sadness. Such a strategy would be akin to the intrapersonal emotion regulation strategy of cognitive reappraisal (Gross, 1998).

The neural basis of intrapersonal emotion regulation is now relatively well-established (see, e.g., Ochsner and Gross, 2005; Wager et al., 2008; Kalisch, 2009; Buhle et al., 2013; Kohn et al., 2014). Such studies have reported a network of brain areas involved in the down-regulation of negative emotion, typically elicited by affective images or videos, including the dorsolateral prefrontal cortex (DLPFC), dorsomedial prefrontal cortex (dmPFC), ventromedial prefrontal cortex (VMPFC), and anterior cingulate cortex (ACC) (Beauregard et al., 2001; Ochsner et al., 2002; Phan et al., 2005; Goldin et al., 2008; Kalisch, 2009). Activity within these frontal regions has been shown to modulate limbic regions such as the amygdala and insula, both of which have been associated with the perception and experience of emotions (Banks et al., 2007; Wager et al., 2008). Supporting evidence is also provided by studies of individuals with serious emotion regulation disturbance, such as major depression and borderline personality disorder, where activation in the frontal cortex and subsequent functional connectivity with limbic regions has been shown to be disrupted during intrapersonal emotion regulation (Johnstone et al., 2007; Koenigsberg et al., 2009).

It has been suggested that people use similar strategies to regulate others' emotions as they use to regulate their own (Niven et al., 2009). However despite the prevalence of interpersonal emotion regulation processes in everyday life (Niven et al., 2009) the neural underpinnings of interpersonal emotion regulation have yet to be directly investigated. A recent study investigated the neural basis of regulating one's own emotion in response to an interpersonal stimulus (namely, a confederate's offer in a bargaining game, Grecucci et al., 2013), finding that reappraisal of another person's intentions recruited areas of frontal cortex, temporo-parietal function and the insula. However, participants were not required to try to regulate the emotional responses of the other person, which is the more typical use of the term interpersonal emotion regulation. The current study builds on this interest in studying the neural basis of interactive elements of emotion regulation, by identifying the neural regions involved in the process of actively regulating another person's emotions, and investigating to what extent this differs or shares similarities with intrapersonal emotion regulation.

Despite overlap in the types of strategy used for intra- and interpersonal emotion regulation (e.g., a person can reappraise their own emotional experience, or help someone else to do the same), the process of interpersonal emotion regulation is likely to differ from intrapersonal emotion regulation in a number of ways. In particular, because interpersonal emotion regulation, by definition, involves an aspect of interpersonal exchange (Zaki and Williams, 2013) it seems likely that elements of social cognition (the processes underlying social perception, engagement and interaction) would be invoked that intrapersonal emotion regulation does not necessarily require. Interpersonal emotion regulation involves the identification of another person's current emotional state, based on contextual cues such as the presence of an emotion eliciting stimulus, as well as bodily information such as facial expression (Zaki and Williams, 2013). Following identification of the other person's emotional state, the affective component of empathy may be needed to mirror an equivalent affective state in the “self” (Preston and De Waal, 2002; Hooker et al., 2010). Once this shared affective state is simulated, the cognitive component of empathy may then be required to take the perspective of the other person. Empathy has been proposed (Preston et al., 2007) to be akin to “theory of mind” or “mentalizing” (Premack and Woodruff, 1978; Gallagher et al., 2000; Frith and Frith, 2003). Finally, monitoring may also be required in order to assess whether the regulation attempt has had the desired effect on the target's emotional state (e.g., does the person look any less emotional?; Zaki and Williams, 2013). Despite these clear conceptual differences between the processes of intra- and interpersonal emotion regulation it is not currently known to what extent the neurophysiological processes supporting the two processes differ.

The present research therefore examined the neural areas activated during interpersonal emotion regulation and intrapersonal emotion regulation. Both forms of emotion regulation were contrasted with a control task where participants were presented with an emotional stimulus but not asked to engage in any form of regulation. We were also interested in whether there would be discernible differences in the neural correlates of interpersonal emotion regulation as a result of the specific strategy used. This follows on from investigations of intrapersonal emotion regulation that have revealed differences in the neural underpinnings of reappraisal and suppression (e.g., Goldin et al., 2008).

We hypothesized that interpersonal emotion regulation would result in overlapping activations with intrapersonal emotion regulation, due to the need to simulate the other person's emotional state and associated regulatory processes. This might involve areas such as the inferior frontal gyrus (BA44; Shamay-Tsoory et al., 2009). Furthermore, we hypothesized that interpersonal emotion regulation would also activate brain areas previously implicated in relevant aspects of social cognition. These include areas involved in perspective taking and “mentalizing,” such as temporo-parietal junction (TPJ), anterior regions of mPFC, superior temporal sulcus (STS) (Gallagher et al., 2000; Vogeley et al., 2001), areas involved in the more cognitive component of empathy (e.g. anterior temporal pole, vmPFC; Shamay-Tsoory et al., 2009), and areas involved in making distinctions between “self” and “other” (rostral frontal cortex; Burgess et al., 2007). Finally, we hypothesized that, in line with previous research (e.g., Goldin et al., 2008; Wager et al., 2008), engaging in intrapersonal emotion regulation would involve a network of frontal regions including DLPFC, VMPFC, and ventrolateral prefrontal cortex (VLPFC), and ACC.

## Materials and methods

### Participants

Twenty right-handed healthy participants (10 males; mean age = 23 years; range 18–30) were recruited from students and staff at a UK University. Exclusion criteria included any psychiatric or neurological disorder or contraindication to MR imaging. All participants spoke English as a first language and had normal or corrected-to-normal vision. Written informed consent was obtained from all participants and the study was approved by the local Research Ethics Committee. Participants were reimbursed for their time.

### Procedure and stimuli

Participants underwent two scans (one intrapersonal and one interpersonal). During each scan, participants viewed a series of short sad or disgusting videos. During each video, participants were instructed to regulate either their own (intrapersonal condition) or another person's (interpersonal condition) emotional state, or simply to watch the video (control “watch” condition). Sadness and disgust were selected as target emotions due to the relative ease of elicitation (Gross and Levenson, 1995). Videos were selected from those used in previous research (e.g., Gross, 1998) and from public access online resources such as YouTube. A pilot study had undergraduate students rate the emotional impact of each video on arousal and the target emotion (disgust or sadness). The final set of videos was matched for arousal rating (mean arousal = 5.47 measured on a 7-point Likert scale).

Each trial commenced with a 5-s introductory screen displaying the title of the video clip (e.g., “child war victims”), the required regulation strategy for that trial (suppress, reappraise, or watch) and three suggested strategy-relevant phrases. Suggested phrases were, for reappraisal; “It's just a film,” “It's not happening to me/you,” or “It's not as bad as it looks,” and for suppression; “Keep a straight face,” “I won't show how I feel”/“Don't show how you feel” or “Grin and bear it.” During the “watch” control condition suggested phrases were “I'll just keep watching”/“You should just keep watching,” “See how this makes me/you feel” or “Watch this carefully.” Participants were asked to select one specific strategy from the list of phrases and to vocalize which strategy they were using during the presentation of the video. This introductory screen was then followed by the 10-s emotion-eliciting video.

**Figure 1:**
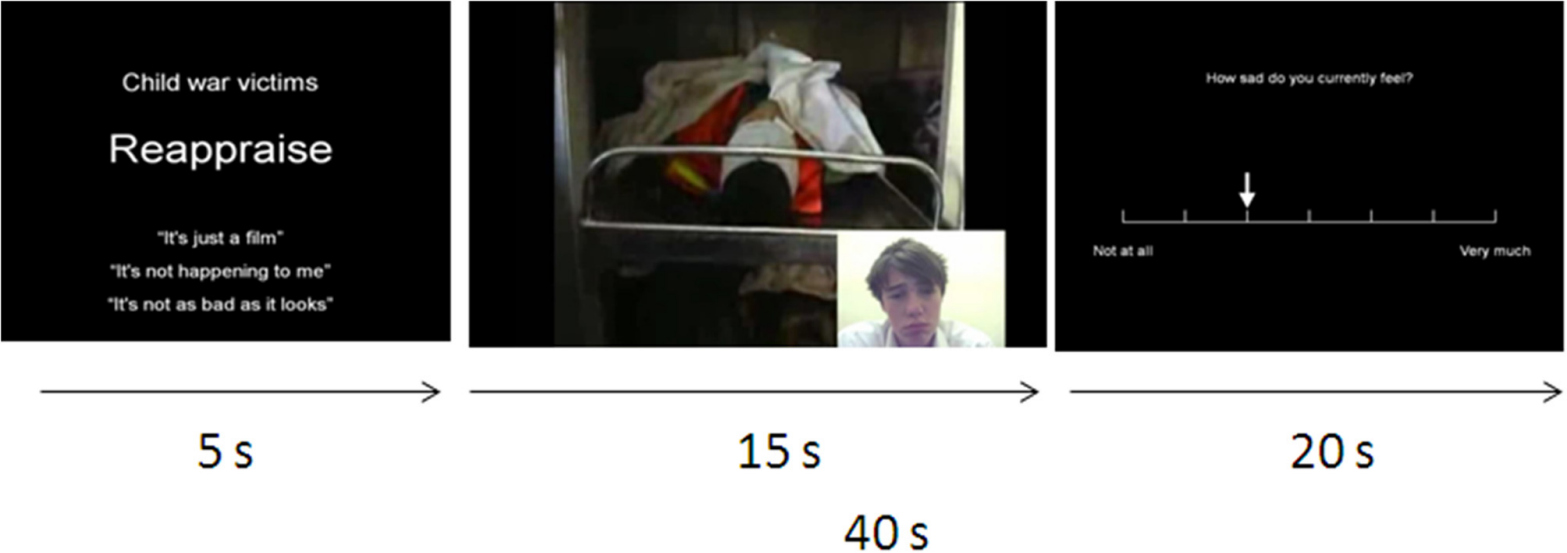
**Description of paradigm**. In both scans, each trial began with the name of the clip and the instruction type (reappraise, suppress, or watch). Three example phrases for emotion regulation were also provided. Following the onset of the video, participants vocalized their choice of regulatory strategy within the first 5 s. At the end of the video, the face of the person watching the videos with them (bottom right-hand corner) remained visible for 5 s. Participants then reported their or the other person's affect and how difficult they found the regulatory process.

During each video, in addition to the emotion-eliciting stimulus, participants also saw another person simultaneously watching and responding to the videos (see Figure 1 for an illustration). This depiction took the form of an embedded video of another person who was visible in the bottom right-hand corner of the screen^1^. For both intra- and interpersonal scans the other person's initial facial expression at the onset of each video was neutral. Following onset of the video, the facial expression changed to one congruent with the content of the video for five seconds (e.g., a disgusted facial expression when the content of the video was disgusting), during which time participants were required to vocalize their emotion regulation strategy^2^. Participants were told that the other person watching the videos would be able to hear them only during the interpersonal run and that they had been instructed to use the advice given by the participant to regulate (or not regulate) their emotional response.

In order to further ensure that participants were engaging in the relevant process (i.e., intra- or interpersonal regulation) participants were told that during the first scan (intrapersonal regulation) the other person would also be focusing on regulating their own emotions. Participants were also told prior to the intrapersonal scan that “During the experiment you will see the face of an actual person in the bottom-right hand corner of the screen. However, we would like the focus of your attention to be on the videos rather than on this person.” Before the inter-personal videos, subjects were told “The second experiment is almost identical to the first. However rather than trying to control your own emotions, we would like you to try to help the other person watching the video to control their emotions. You have already seen these videos and so have experienced how to control your own emotions. We now want you to use this experience to tell the person in the corner of the screen how they should control their emotions.”

For the next 5 s of the video, following the vocalization of a strategy, participants were required to continue watching the video and continue to regulate their emotions, or those of the other person using their chosen regulation strategy. During this period, on two-thirds of the trials the facial expression of the other person began to slowly return to neutral. On the other one-third of the trials, the facial expression of the other person did not return to “neutral,” but stayed as the target emotion, suggesting that the participant's regulatory effort had been apparently ineffective on that trial. This procedure was adopted to reflect the fact that intra- and interpersonal regulation are not always successful in everyday life^3^. As the participant spoke during the first 5 s following onset of the video, it therefore appeared that the participants' utterance in the interpersonal condition influenced the other person's emotional response. In other words, the regulatory attempt and the other person's emotional response were temporally bound together in a manner that promoted a causal interpretation. On some videos the other person would occasionally make eye contact with the camera, and make small gestures (e.g., head nodding, etc.) toward the camera in order to give the sense that the interaction between the participant and themselves was dynamic. During production of the videos of the other person they had been watching the actual videos on a monitor and heard sample regulatory phrases spoken during the time-frame in which the participants would subsequently be doing so. Therefore, the reactions were matched to the content and time-course of the videos. Following the video there was then another 5-s period during which participants continued to implement their emotion-regulation strategy and continued to see only the other person watching the video with them in the corner of the screen. During this time the facial expression of the other person continued to return to neutral (or remained as the target emotion in one-third of trials).

In order to measure the effects of implementing emotion regulation strategies on emotional arousal and effort, during the final 20-s of each trial participants used a button box to rate, on a 7-point Likert scale, the intensity of their own or the other person's emotional experience (“how disgusted / sad do you currently feel / how disgusted / sad does *person outside the scanner* feel?”), and how difficult they found that trial (“how difficult did you find it to follow the instructions?”; see Figure 1). These measures also ensured that participants were adhering to the task and making effortful attempts to follow the instructions on each trial.

Both the intra- and interpersonal scans consisted of 21 trials that each involved watching a video, enacting emotion regulation (or watch) strategy and answering self-report questions. Within each scan three sadness-inducing and three disgust-inducing video clips were viewed three times each, under instructions to “watch,” “reappraise,” or “suppress.” Additionally, three “neutral” videos were viewed under the condition of “watch” only. The order of the videos was pseudo-randomized such that no regulation strategy (suppress, reappraise, watch) or type of video (disgusting, sad, neutral) appeared more than three times in succession. Four different orders of the stimuli within each scan were used in a between-participant counter-balanced design. At the end of all the scanning sessions participants were debriefed.

In experiments investigating interpersonal interactions such as interpersonal emotion regulation, it is important to establish that the participants are meaningfully engaging with each other. To this end, within this study, the person in the video had met with the participant on the day prior to the scan and conducted a practice session with them. This ensured that each of the participants had the same level of interaction and familiarity with the other person, and also increased the likelihood that participants would be motivated to regulate that person's emotion. The practice session also ensured that prior to the scanning sessions participants were familiar with the tasks and nature of the videos they would be seeing. In summary, and importantly for interpreting differences between the intra- and interpersonal tasks, the design ensured that the two conditions were identical in all respects and differed only in the perspective that the participant in the scanner adopted; i.e., focusing either on regulating their own or another person's response to an emotionally arousing video.

### fMRI data acquisition

During each functional run, 280 time points were obtained at 3T (Achieva, Philips Medical Systems, Best, NL) comprising 32 × 4 mm thick contiguous slices (in-plane resolution 1.797 × 1.797 mm) covering the entire cerebrum and cerebellum. A single-shot, gradient-recalled echo planar imaging (EPI) sequence was used: repetition time = 3 s; echo time = 35 ms; FOV = 240 mm; in-plane matrix = 128 × 128 mm). A high resolution, whole-brain, T1-weighted structural scan was also collected from each participant (3D gradient echo, MP-RAGE, *TR* = 10.5 ms; *TE* = 4.8 ms; spatial resolution = 0.8 mm^3^).

During the scan participants wore MR-compatible headphones with microphone that was adjusted to fit over the mouth. Participants were informed of the importance of minimizing head movement. Specifically, they were told that the microphone was sensitive and they therefore did not need to speak too loudly while providing the regulatory phrase. This helped ensure to that participants' head movements were kept to a minimum during the scan.

### fMRI data preprocessing

Scan data were analyzed in SPM 8 (http://www.fil.ion.ucl.ac.uk/spm/) implemented in MATLAB 7.1 (Math-works Inc., Sherborn, MA). Images were motion-corrected, spatially normalized to each individual's high-resolution T1-weighted scan, and smoothed with a Gaussian kernel (full-width half-maximum of 8 mm). Two out of the total 40 functional runs were discarded due to excessive head motion during the scan (>2.5 mm in any plane). Blood-oxygen-level-dependent (BOLD) response was modeled to an event related wave-form, convolved with a canonical hemodynamic response function and its temporal derivative. Participants' movement parameters were included as regressors in the final contrast model in order to control for movement artifacts.

Our contrast of interest focused on the 15-s period where participants watched the video and performed emotion regulation. At the first individual subject-level, epochs of “reappraisal” and “suppression” were contrasted with emotional videos viewed under the instruction to “watch.” These first level, fixed-effects analyses were taken forward to a second, group-level, flexible factorial design, that allowed examination of main-effects and interactions, with factors of participant, scan (intrapersonal or interpersonal) and regulation strategy (reappraise, watch, or suppress). Results are presented at *p* < 0.001 uncorrected. This statistical threshold was employed given the exploratory and novel nature of these comparisons and is in line with recommendations for such complex and subtle cognitive processes, as used in previous social and affective neuroscience studies (Lieberman and Cunningham, 2009).

## Results

### Effect of intrapersonal and interpersonal emotion regulation on self-report behavioral ratings

**Figure 2:**
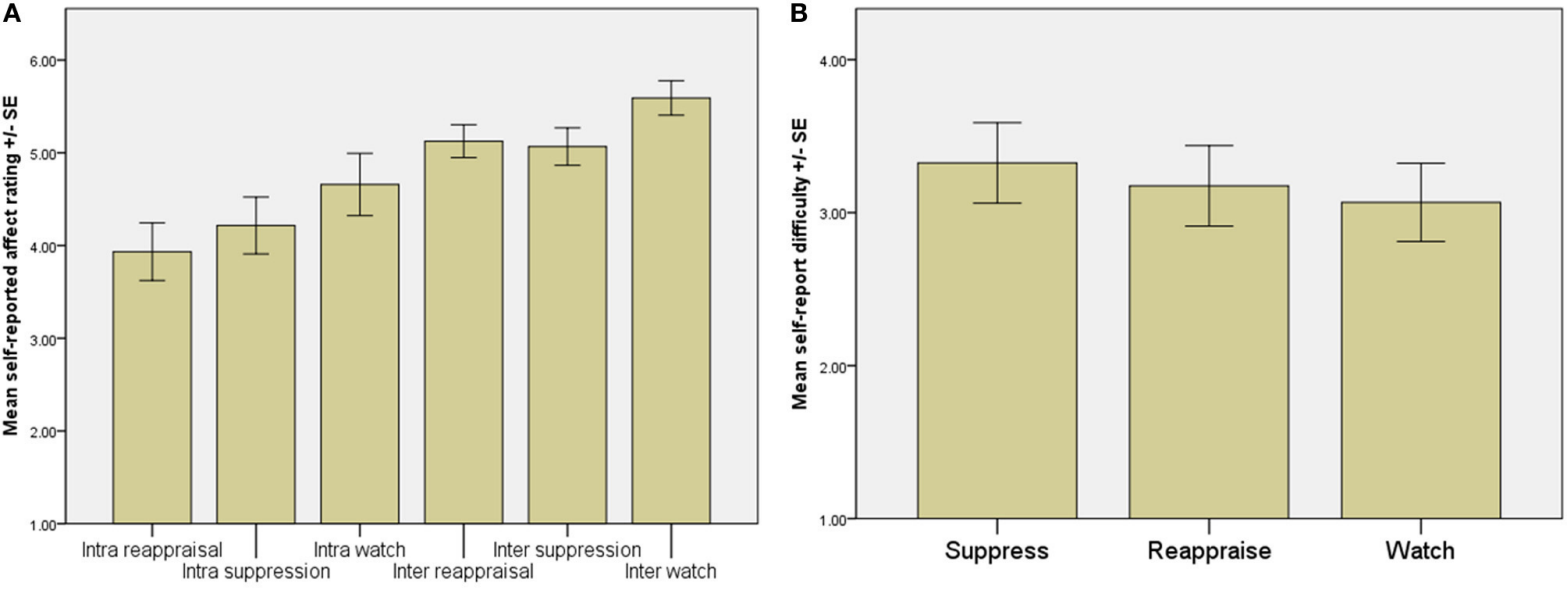
**(A)** Mean ratings for how emotional the participants felt (intrapersonal scan) or how emotional the participant judged the other person to be (interpersonal scan). There was a main effect of regulation strategy [*F*_(2, 38)_ = 8.04, *p* < 0.01]. For intra- and interpersonal scans, emotions were higher in the watch condition in comparison to both the suppression (*p* < 0.05, *p* < 0.001 for intra- and interpersonal regulation, respectively) and reappraisal (*p* < 0.01, *p* < 0.001) conditions. During intrapersonal regulation, ratings were also significantly higher for suppression in comparison to reappraisal trials (*p* < 0.05) but not for interpersonal regulation. Ratings for the “intra” conditions refer to the participant's current affective state, whereas ratings for the “inter” conditions refer to what the participant judged the other person's affective state to be. **(B)** There was no main effect of regulation type on the difficulty of following instructions in the interpersonal regulation run [*F*_(2, 38)_ = 0.55, *p* = 0.58], indicating that participants adhered to instructions and engaged in the relevant processes.

A 3-within scan (regulation strategy: suppression, reappraisal or watch) by 2-between scan (regulation type: intrapersonal or interpersonal) repeated measures ANOVA with emotional experience as the dependent variable showed a main effect of regulation strategy [*F*_(2, 38)_ = 8.04, *p* < 0.01, Figure 2] with emotional experience being higher in the watch condition than in the suppression (*p* < 0.05, *p* < 0.001 for intra- and interpersonal, respectively) and reappraisal (*p* < 0.01, *p* < 0.001) conditions. For the intrapersonal scans, ratings of emotional experience were also significantly higher for suppression than for reappraisal trials (*p* < 0.05), suggesting that suppression was a less effective strategy for regulating own, but not others, emotions. There was no main effect of regulation type (intra- vs. inter-personal) on the perceived effort expended [*F*_(2, 38)_ = 0.55, *p* = 0.58, Figure 2].

### Neurophysiological basis of intrapersonal emotion regulation

**Figure 3:**
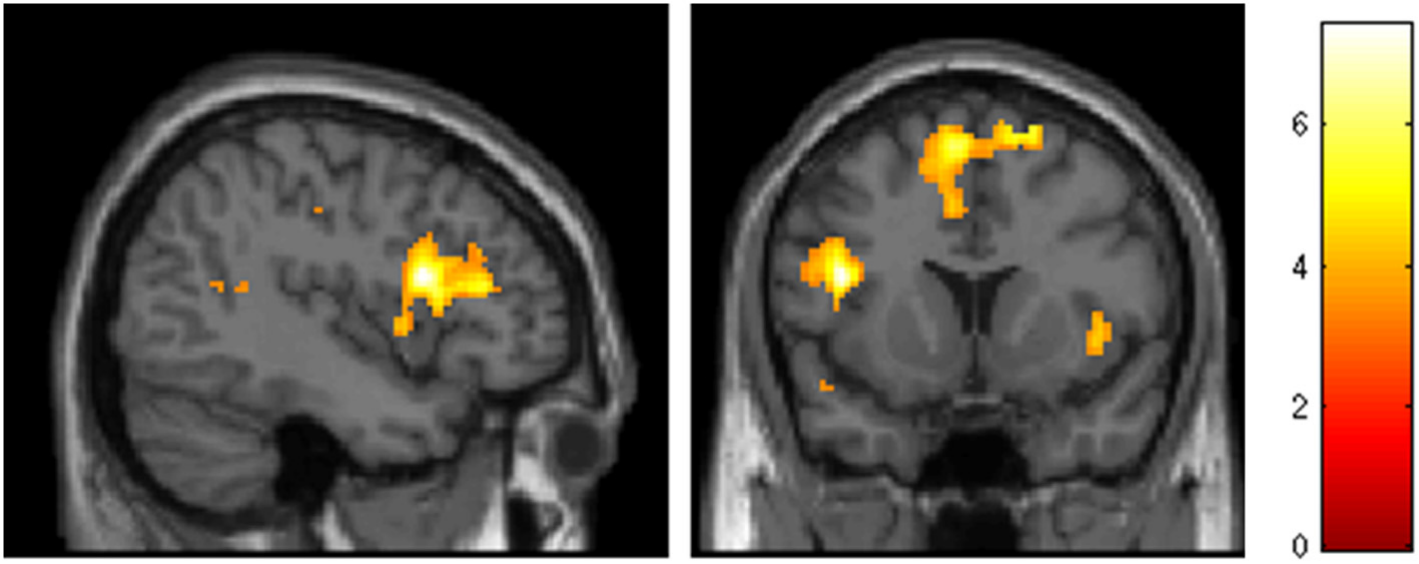
**Intrapersonal emotion regulation (reappraisal & suppression vs. watch), *p* < 0.001, extent threshold = 10 voxels**.

Intrapersonal regulation (reappraisal and suppression combined) contrasted with watch trials was associated with activation of left IFG (BA 45), pSMA (BA 6), right DLPFC (middle frontal gyrus; BA 46), right VLPFC (BA 47), left superior frontal gyrus (BA 10), right supramarginal gyrus/TPJ, left posterior cingulate, left TPJ (BA 39/40), right cuneus/posterior cingulate (BA 23/31), and cerebellum (*p* < 0.001 uncorrected; Table 1, Figure 3).

**Table 1:** **Areas activated in the contrast intrapersonal emotion regulation (reappraisal and suppression combined) > watch**.

**Area**	**Tal coordinates**			**Voxels**	***z* value**
	***X***	***Y***	***Z***		
Lt. Inferior frontal gyrus (BA 45)	−42	12	16	681	5.75
	*−44*	*30*	*13*		*4.52*
	*−42*	*6*	*3*		*3.76*
Pre-supplementary motor area (BA 6)	−2	13	56	824	5.13
	*22*	*11*	*58*		*4.83*
	*14*	*15*	*58*		*4.57*
Lt. Posterior cingulate gyrus	−18	−38	24	61	4.53
Lt. Temporo-parietal junction	−48	−44	21	386	4.40
	*−48*	*−53*	*19*		*4.35*
	*−59*	*−52*	*−39*		*3.78*
Lt. Cerebellum	−6	−56	−39	138	4.36
/ *Peri-acqueductal gray*	*6*	*−45*	*−36*		*4.18*
	*2*	*−52*	*−39*		*3.89*
Rt. Dorsolateral prefrontal cortex	44	36	24	322	4.27
(BA 46)	*38*	*40*	*18*		*3.80*
	*36*	*41*	*9*		*3.61*
Rt. Insula (BA 13)	42	10	0	128	4.24
Rt. Ventrolateral prefrontal cortex (BA 47)	32	19	−6		4.02
Rt. Temporo-parietal junction /supramarginal gyrus	36	−49	32	109	4.23
Lt. Superior frontal gyrus (BA 10)	−28	40	15	120	4.20
Bilateral cuneus /post. cingulate (BA 23/31)	14	−61	18	610	4.14
	*−8*	*−68*	*33*		*4.03*
	*8*	*−63*	*31*		*3.92*

*Data presented at p < 0.001 (uncorrected) with an extent threshold of 30 voxels*.

*Co-ordinates are shown in standardized neuroanatomical space (Talairach and Tournoux, 1988). BA, Brodmann's area; Lt., left; Rt., right; post., posterior. Co-ordinates without a corresponding extent threshold are shown in italics and refer to sub-clusters of the preceding activation*.

### Neurophysiological basis of interpersonal emotion regulation

**Figure 4:**
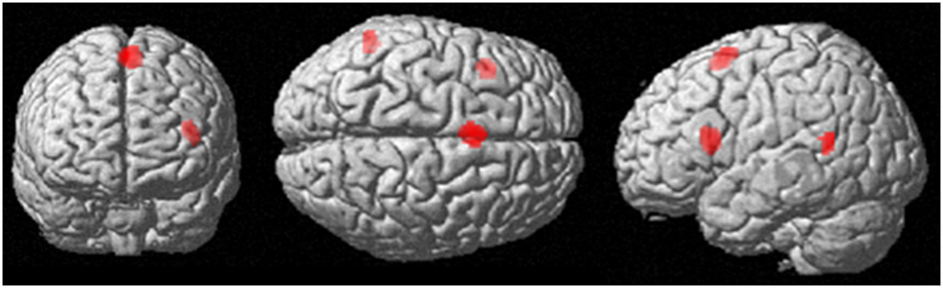
**Conjunction analysis showing common activations of intrapersonal and interpersonal emotion regulation, *p* < 0.001, extent threshold 30 voxels**.

Interpersonal emotion regulation (reappraisal and suppression combined) contrasted with “watch” trials was associated, in common with intrapersonal emotion regulation, with activation of IFG (BA 45) and pSMA (BA 6); but additionally with activation within bilateral inferior temporal gyrus (BA 20), rostral medial prefrontal cortex (mPFC; BA 10), mPFC (BA 8 and 9), left ACC (BA 9/32), left TPJ (BA 39/40) and right temporal pole; (BA38) (*p* < 0.001, Table 2, Figure 4).

**Table 2:** **Areas activated in the contrast interpersonal emotion regulation (reappraisal and suppression combined) > watch**.

**Area**	**Tal coordinates**			**Voxels**	***z* value**
	***X***	***Y***	***Z***		
Lt. Inferior frontal gyrus (BA 45)	−38	16	14	109	5.32
Pre-supplementary motor area (BA 6)	−4	11	57	163	4.98
Rt. Inferior temporal gyrus (BA 20)	50	−7	−28	58	4.91
	*48*	*−2*	*−34*		*3.55*
Rostral medial prefrontal cortex (BA 10)	4	59	12	155	4.40
Rt. Superior frontal gyrus (BA 10)	16	59	17	120	3.96
	*24*	*57*	*14*		*3.61*
Rt. Medial prefrontal cortex (BA 9)	14	44	18	85	4.36
	*16*	*43*	*5*		*4.17*
Lt. Anterior cingulate (BA 9/32)	−10	38	17	63	4.26
Lt. Inferior temporal gyrus (BA 20)	−44	−13	−21	34	4.13
	*−44*	*−18*	*−16*		*3.39*
Medial frontal gyrus (BA 8)	2	31	39	73	4.08
	*4*	*23*	*38*		*3.39*
Pulvinar	−4	25	14	76	4.03
Lt. Temporo-parietal junction	−61	−48	12	156	3.92
	*−53*	*−44*	*13*		*3.78*
	*−58*	*−45*	*26*		*3.72*
Rt. Temporal pole (BA 38)	48	15	16		3.82
	*51*	*17*	*−8*		*3.69*
	*57*	*18*	*1*		*3.30*

*Data presented at p < 0.001 (uncorrected) with an extent threshold of 30 voxels*.

*Co-ordinates are shown in standardized neuroanatomical space (Talairach and Tournoux, 1988). BA, Brodmann's area; Lt., left; Rt., right; post., posterior. Co-ordinates without a corresponding extent threshold are shown in italics and refer to sub-clusters of the preceding activation*.

### Common activations for intrapersonal and interpersonal emotion regulation

**Figure 5:**
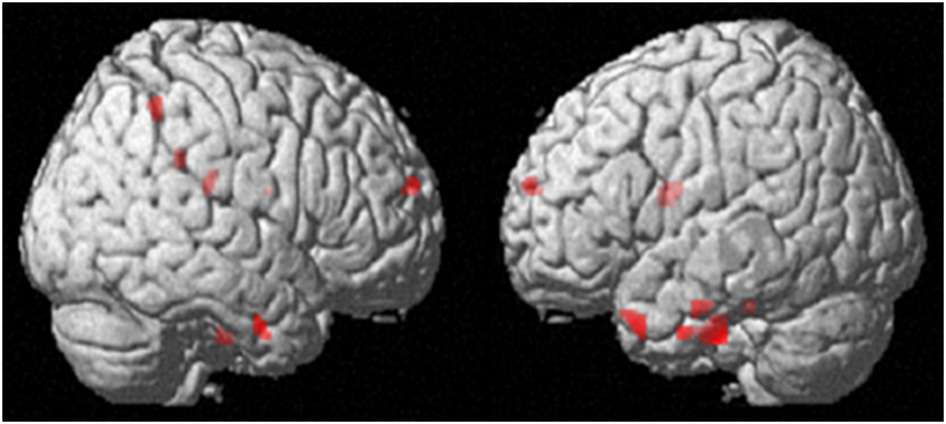
**Interpersonal emotion regulation (interpersonal reappraisal & suppression vs. watch), *p* < 0.001 uncorrected, extent threshold = 10 voxels**.

A conjunction analysis was performed to further investigate brain areas involved in both the intrapersonal and interpersonal conditions. This revealed areas involved in both conditions were the pSMA (BA6), left IFG (BA45), and the left TPJ (BA39/40) (Table 3, Figure 5).

**Table 3:** **Conjunction analysis showing areas involved in both intrapersonal and interpersonal conditions**.

**Area**	**Tal coordinates**			**Voxels**	***z* value**
	***X***	***Y***	***Z***		
Lt. Inferior frontal gyrus (BA 45)	−38	18	14	91	5.14
Pre-supplementary motor area (BA 6)	−4	11	57	128	4.89
Lt. Temporo-parietal junction	−59	−50	14	56	3.67

*Data presented at p < 0.001 (uncorrected) with an extent threshold of 30 voxels*.

*Co-ordinates are shown in standardized neuroanatomical space (Talairach and Tournoux, 1988). BA, Brodmann's area; Lt., left; Rt., right; post., posterior*.

### Comparison of the neurophysiological bases of intrapersonal and interpersonal emotion regulation

Brain areas more involved in intrapersonal than interpersonal emotion regulation included bilateral posterior cingulate (BA 31), right ACC (BA 32), right insula (BA 13), left DLPFC; BA 46), left precentral gyrus/inferior frontal gyrus (BA 6/9), left mPFC (BA 6), right cerebellum, left superior temporal gyrus (BA 22), and left superior frontal gyrus (Table 4). Areas more involved in interpersonal than intrapersonal emotion regulation included the left temporal pole (superior temporal gyrus; BA 38), rostral mPFC (BA 10), bilateral inferior temporal gyrus (BA 20), right posterior insula (BA 13), right cingulate gyrus (BA 31), bilateral caudate, and right cuneus/inferior parietal lobule (BA 7/40) (Flexible factorial design, Table 5).

**Table 4:** **Areas activated by the main effect of intrapersonal emotion regulation (intrapersonal emotion regulation - watch > interpersonal emotion regulation > watch)**.

**Area**	**Tal coordinates**			**Voxels**	***z* value**
	***X***	***Y***	***Z***		
Rt. Posterior cingulate gyrus	28	−51	19	193	4.17
(BA 31)	*26*	*−53*	*36*		*3.05*
	*20*	*−56*	*16*		*2.63*
Lt. Posterior Cingulate gyrus	−18	−38	24	35	3.63
Peri-acqueductal gray	4	−46	−33	75	3.45
	*−2*	*−56*	*−39*		*3.29*
	*6*	*−54*	*−33*		*2.79*
Rt. Anterior Cingulate (BA 32)	22	30	19	50	3.43
Rt. Insula (BA 13)	44	10	0	33	3.40
	*36*	*8*	*−4*		*2.94*
Lt. Dorsolateral prefrontal cortex (BA 46)	−42	30	11	36	3.40
Lt. Precentral gyrus (BA 6/9)	−48	−7	24	32	3.31
Lt. Medial frontal gyrus (BA 6)	−8	−26	53	32	3.22
	*−18*	*11*	*58*	*37*	*3.19*
Rt. Cerebellum	30	−42	−28	79	3.18
	*36*	*−42*	*−21*		*2.82*
Lt. Superior temporal gyrus (BA 22)	−55	−10	4	50	3.16
	*−55*	*−7*	*11*		*3.11*
Lt. Superior frontal gyrus	−28	40	15	36	3.06

*Data presented at p < 0.001 (uncorrected) with an extent threshold of 10 voxels*.

*Co-ordinates are shown in standardized neuroanatomical space (Talairach and Tournoux, 1988). BA, Brodmann's area; Lt., left; Rt., right; post., posterior. Co-ordinates without a corresponding extent threshold are shown in italics and refer to sub-clusters of the preceding activation*.

**Table 5:** **Areas activated by the main effect of interpersonal emotion regulation (interpersonal emotion regulation - watch > intrapersonal emotion regulation > watch)**.

**Area**	**Tal coordinates**			**Voxels**	***z* value**
	***X***	***Y***	***Z***		
Lt. Temporal pole (BA 38)	−34	14	−28	34	4.30
Lt. Pons	−16	−21	−28	77	3.80
Rostral medial PFC(BA 10)	2	59	15	18	3.48
Lt. Inferior temporal gyrus (BA 20)	−44	−13	−21	11	3.44
Rt. Posterior Insula (BA 13)	28	−24	21	10	3.20
Lt. Caudate	−10	1	15	27	3.11
Rt. Inferior temporal gyrus (BA 20)	51	−7	−27	12	3.09
Pons	−2	−32	−19	20	3.07
Rt. Cuneus/Inferior parietal lobule (BA 7/40)	26	−44	48	17	2.91

*Data presented at p < 0.001 (uncorrected) with an extent threshold of 10 voxels*.

*Co-ordinates are shown in standardized neuroanatomical space (Talairach and Tournoux, 1988). BA, Brodmann's area; Lt., left; Rt., right; post., posterior*.

We also conducted an exploratory comparison of whether the activations observed for interpersonal regulation differed according to the instruction of reappraisal and suppression, though no hypotheses had been made about this given that there have been no previous investigations into the neural basis of different interpersonal emotion regulation strategies, and there was no self-reported difference between perceived effectiveness of each strategy from the self-report data.

### Interpersonal reappraisal

The contrast of interpersonal reappraisal emotion regulation trials contrasted with interpersonal watch trials revealed activation within rostral prefrontal cortex (BA10), medial prefrontal cortex (BA8), left temporal pole (BA38), and left insula (Table 5). Expanding this contrast to directly compare interpersonal and intrapersonal reappraisal revealed increased activation in left inferior temporal gyrus (BA20), left temporal pole (BA38), left putamen, rostral medial prefrontal cortex (BA10), left cingulate gyrus (BA23), and right inferior parietal lobule (Table 6).

**Table 6:** **Areas activated by interpersonal emotion regulation**.

**Area**	**Tal coordinates**			**Voxels**	***z* value**
	***X***	***Y***	***Z***		
**INTERPERSONAL REAPPRAISAL > WATCH**					
Rt. Superior frontal gyrus (BA8)	14	33	46	13	4.07
Medial prefrontal cortex (BA10)	4	59	10	77	3.98
	*8*	*51*	*7*		*3.63*
	*8*	*49*	*14*		*3.26*
Rt. Caudate	14	−24	18	16	3.79
Lt. Putamen	−22	−4	7	25	3.58
Lt. Temporal Pole (BA38)	−55	14	3	14	3.50
Lt. Cuneus (BA7)	−14	−58	53	15	3.45
Thalamus	4	−21	3	18	3.45
Peri-acqueductal gray	2	−35	−3	21	3.38
**INTERPERSONAL SUPPRESSION > WATCH**					
Lt. Inferior temporal gyrus (BA20)	−50	−7	−28	31	4.77
	*−46*	*−9*	*−18*		*3.31*
Medial prefrontal cortex (BA8)	2	31	39	57	4.62
Lt. Inferior frontal gyrus (BA45)	−40	20	14	70	4.34
Rt. Superior frontal gyrus (BA6)	18	16	53	29	4.29
Rt. Anterior insula (BA13)	36	16	0	35	4.15
Lt. Cingulate gyrus (BA32)	−10	21	32	25	4.11
Lt. TPJ (BA40)	−57	−47	24	35	3.93
Rt. Middle frontal gyrus (BA9)	34	27	37	41	3.81
Medial prefrontal cortex (BA10)	16	43	5	21	3.55
**INTERPERSONAL REAPPRAISAL > INTRAPERSONAL REAPPRAISAL**					
Lt. Inferior temporal gyrus (BA20)	−38	−17	−21	26	4.27
Lt. Pons	−8	−17	−23	21	3.90
Lt. Putamen	−24	−11	13	31	3.88
Lt. Temporal Pole (BA38)	−36	10	−27	28	3.85
Rt. Caudate	26	−22	23	20	3.84
Medial prefrontal cortex (BA10)	8	51	7	32	3.81
Lt. Cingulate gyrus (BA23)	−10	−14	28	23	3.74
Lt. Superior frontal gyrus (BA9)	−6	50	23	16	3.63
Rt. Inferior parietal lobule (BA7)	30	−46	47	25	3.56
**INTERPERSONAL SUPPRESSION > INTRAPERSONAL SUPPRESSION**					
Lt. Anterior temporal pole (BA38)	−34	16	−26	14	4.08
Lt. Inferior temporal gyrus (BA20)	−50	−7	−28	6^*^	3.94
Rt. Lingual gyrus	20	−84	−8	8^*^	3.50
Lt. Caudate	−8	1	17	13	3.43

Data presented at p < 0.001 (uncorrected) with an extent threshold of 10 voxels (

* *indicates clusters with an extent threshold of 5 voxels)*.

*Co-ordinates are shown in standardized neuroanatomical space (Talairach and Tournoux, 1988). BA, Brodmann's area; Lt., left; Rt., right; post., posterior. Co-ordinates without a corresponding extent threshold are shown in italics and refer to sub-clusters of the precedingactivation*.

### Interpersonal suppression

The contrast of interpersonal suppression emotion regulation trials contrasted with interpersonal watch trials revealed activation within left inferior temporal gyrus/temporal pole, medial prefrontal cortex (BA8), left inferior frontal gyrus, right anterior insula (BA13), superior frontal gyrus (BA6), left cingulate gyrus (BA32), and left temporo-parietal junction (BA40). Expanding this contrast to directly compare interpersonal and intrapersonal suppression revealed increased activation in the left anterior temporal pole (BA38) and left caudate (Table 6).

### Post scanning debriefing

To clarify the experiences of participants during the experiment we debriefed participants following the scans. Participants believed that the phrases they had uttered during the interpersonal scan had been used by the conspecific to try and control their emotional responses. No participants reported any suspicion that the experimental set-up had not been live.

## Discussion

The present research directly compared the regulation of own emotions (intrapersonal emotion regulation) with the regulation of another person's emotions (interpersonal emotion regulation). Both types of regulation recruited areas previously implicated in intrapersonal regulation, including inferior frontal gyrus and pre-supplementary motor area. However interpersonal regulation, which involves helping another person to reappraise or suppress their emotions, additionally recruited areas that have been previously implicated in mentalizing and other facets of social cognition, including the left anterior temporal pole and medial prefrontal cortex.

Both intrapersonal and interpersonal emotion regulation were associated with a largely overlapping network of brain areas, incorporating the bilateral lateral frontal cortices, the pSMA, and left TPJ. Frontal activation is consistent with the findings of previous studies on the cognitive control of one's own emotion (Ochsner and Gross, 2005; Banks et al., 2007; Wager et al., 2008; Kalisch, 2009) and suggests that both tasks involved a degree of control over one's own emotional experience. This was in line with our hypotheses for the intrapersonal task. However finding similar areas of activation for the interpersonal condition supports the notion that successfully regulating another person's emotion involves “simulation” of the intrapersonal process. This simulation may, in part, explain why interpersonal emotion regulation has been considered an “effortful” process that affects social and personal well-being (Niven et al., 2012).

It is noteworthy that we found activation within the pSMA for both conditions (as confirmed by conjunction analysis). A meta-analysis by Kalisch (2009) of the regulation of negative emotion by reappraisal reported that activation within pSMA is a consistent finding across studies. One possible explanation is that activation within pSMA reflects the requirement for response inhibition (Hampshire et al., 2010); something that is an inherent component of emotion regulation. It is also possible that, given this area's involvement in occulomotor control (Grosbras et al., 2005), activation within pSMA may reflect the redeployment of attentional resources to non-emotional components of a stimulus (van Reekum et al., 2007). Further studies could attempt to clarify the precise role of the pSMA in emotion regulation. Such an investigation would be highly informative given the consistency with which this region has been activated, not only within studies of emotion regulation but also within neuroimaging studies more generally (Behrens et al., 2013).

Although the involvement of TPJ in the interpersonal condition was hypothesized (given its previously identified role in mentalizing, (e.g., Gallagher et al., 2000), TPJ has not been consistently associated with paradigms involving the control of one's own emotional experience. One speculative explanation for the role of TPJ in intrapersonal emotion regulation is that our study conceptualized intrapersonal emotion regulation in a more social context. That is, the task involved the control of emotion in the presence of another person, albeit a person that the participant was instructed to ignore. It is possible, that participants had the sense that their emotion regulation was in part motivated (and perhaps being monitored) by the other person, and so processes akin to mentalizing were evoked (i.e., “what does the other person think of my regulation attempts?”). This explanation is consistent with the traditional view that the TPJ underpins such mentalizing processes (Gallagher and Frith, 2003), and is consistent with a recent study that found TPJ involvement in the reappraisal of another person's intentions (Grecucci et al., 2013).

The left DLPFC and ACC were significantly activated in the intrapersonal regulation task when compared directly with the interpersonal condition. Activation within the frontal cortex, particularly DLPFC, is consistent with the existing literature on the cognitive control of emotion (Beauregard et al., 2001; Ochsner et al., 2002; Phan et al., 2005; Eippert et al., 2007; Goldin et al., 2008). Activation in the cuneus/posterior cingulate was not hypothesized during intrapersonal regulation; however, one plausible interpretation would be that this region is involved in self-reflective processing (Lou et al., 2004) and the instructions for intrapersonal regulation required participants to focus on their own rather than the other person's emotions. Activation within the posterior cingulate may therefore reflect the increased requirement for self-monitoring during the intrapersonal task, whereas in the interpersonal condition there was no requirement to self-monitor. Participants also knew that they would be asked to reflect upon their own emotional state during the questions that followed each trial during the intrapersonal run.

### The neurophysiology of interpersonal emotion regulation

Brain areas uniquely involved in interpersonal regulation included the left temporal pole, rostral medial prefrontal cortex, bilateral inferior temporal gyrus, right posterior insula, right cingulate gyrus, bilateral caudate, and right cuneus/inferior parietal lobule. When this activation was decomposed into the neural regions associated with reappraisal or suppression there were activations common to both strategies and also some differences. Both strategies showed increased activation within left anterior temporal pole and inferior temporal gyrus. However, interpersonal reappraisal was also associated with additional activation of rostral prefrontal cortex and areas of cingulate gyrus. One explanation for this additional activation might be that reappraisal requires a clearer distinction to be made between the self and another person than does suppression. Such an explanation is supported by the posited role of rostral prefrontal cortex and areas of cingulate gyrus in supporting distinctions between the self and others (Burgess et al., 2007; Raposo et al., 2011), although the medial prefrontal cortex has also been shown to be involved in representation of the self (Qin and Northoff, 2011; Denny et al., 2012). Activation within rostral PFC has also been reported as reflecting the emotional synchrony between two individuals (Kühn et al., 2011); something that participants were likely to be more engaged in during interpersonal regulation than during intrapersonal regulation. The increased activation within rostral PFC during interpersonal emotion regulation using reappraisal suggests that this emotional synchrony may have been more pronounced for reappraisal than for suppression, because of the more cognitive nature of reappraisal in comparison to the more behavioral instruction of suppression.

We also found involvement of the caudate during interpersonal reappraisal. Although this had not been hypothesized, case studies have suggested that damage to the caudate impairs the affective components of theory of mind and recognition of other people's negative emotional states (Kemp et al., 2013), both of which are likely to be involved in helping another person to reappraise their emotions. Furthermore given that the caudate has also been shown to be activated when a reward is expected (Haruno et al., 2004; Benningfield et al., 2014) it is also possible that the activation of the caudate may reflect a sense of reward felt by participants at helping the other person control their emotion, given that interpersonal emotion regulation is known to have effects not only on the target of the regulation but also on the person doing the regulation (Niven et al., 2012). Such an explanation might be consistent with studies that have suggested that caudate is particularly involved in processing reward that arises as a direct result of the participant's actions (Tricomi et al., 2004; see Grahn et al., 2008 for a review of the role of the caudate in cognition). Interpersonal suppression, when compared to watch, also revealed activation in the right anterior insula. This is noteworthy given the insula's involvement in the representation of emotional experience (Zaki et al., 2012) and in affective decision making (Singer et al., 2009), and could further support the notion that participants simulated the other person's affective state during interpersonal emotion regulation.

Activation within the anterior temporal pole during interpersonal emotion regulation is consistent with studies that demonstrate the involvement of these areas in cognitive empathy such as “mentalizing” (Gallagher and Frith, 2003) and the interpretation of another person's mental state (Jimura et al., 2010). These findings support the idea that interpersonal emotion regulation is also underpinned by similar processes to mentalizing, which is consistent with the need to take the perspective of the other person in order to successfully regulate their emotion.

Activation within the bilateral inferior temporal gyri has also been reported in previous research into the neural basis of empathy (Farrow et al., 2001), although this activation has been less consistently observed than has activation in areas such as the medial prefrontal cortex. Activation within the bilateral inferior temporal gyri within the current study supports this region's contribution to empathy. However, given that we had made no specific hypothesis concerning this region, this finding must be treated with caution. Activation within areas of anterior temporal pole for both conditions (interpersonal reappraisal and suppression), taken in conjunction with activation of inferior frontal gyrus associated with the regulation of a person's own emotional state, may also lend support to the affective simulation account of empathy (Decety and Lamm, 2007). Activation in the inferior frontal gyrus suggests that regulating another person's emotion may involve “reliving” the emotional response and modeling the regulation process in order to help the other person to control their emotional state.

### Limitations

One limitation of the current study is that, although providing advice on how to regulate emotions to another person and monitoring the effects of the advice does constitute one form of interpersonal emotion regulation, there are nevertheless many other forms of interpersonal emotion regulation that differ in their manner of implementation and intended effects (for a review, see Niven et al., 2009). One dimension on which types of interpersonal emotion regulation may differ concerns an individual's reasons for performing interpersonal emotion regulation, which may vary from the entirely selfish to the wholly altruistic. For example, an individual in a relationship may selfishly wish to quickly regulate their partner's negative emotion in order to allow them to watch their favorite TV program. It seems likely that more selfish reasons for performing emotion regulation may not necessarily invoke the mechanisms of empathy to the same extent as seen in this study. Further studies could investigate this issue by, for example, investigating regulating the emotions of someone better known to the participant (e.g., a close friend) would result in a different pattern of activation to regulating the emotions of a less well-known person, as in the present research. Further studies may also wish to further explore the differences between successful and unsuccessful interpersonal regulation attempts in more detail.

Another limitation of the current study was that the intrapersonal scan always preceded the interpersonal scan, leaving open the possibility that activations, particularly within the interpersonal scan, may be influenced to some extent by habituation to the stimuli or repetition of the general regulatory processes. Debriefing of participants during piloting of the paradigm prior to scanning revealed that it was not possible to counterbalance the order of runs in such a manner that participants were able to fully engage in interpersonal regulation without first having engaged in intrapersonal regulation. Future research could, however, focus exclusively on a more in-depth exploration of interpersonal regulation. Alternatively, studies might consider using a different set of emotion-eliciting stimuli in an intrapersonal task, as compared to an interpersonal task. We felt that repetition of the stimuli was necessary in the present research, given the nuanced nature of our paradigm and the need to keep as much as possible equivalent between intrapersonal and interpersonal emotion regulation. Specifically, we wanted to use the same videos in the interpersonal run as in the intrapersonal run to focus participants' attention on the interpersonal regulation aspect; essentially, the participant was using their experiences from the intrapersonal scan in order to help the other person in the interpersonal scan.

Finally, the presence of the other person in the corner of the screen in the intrapersonal task may have interfered with the self-regulation process because participants may have been monitoring the other person in addition to attempting to regulate their own emotional response. This is reflected in the data to the extent that regions associated with social cognition, such as TPJ, were activated during intrapersonal emotion regulation. However, the fact that (i) we found activation within regions previously demonstrated to be involved in intrapersonal regulation (pSMA, DLPFC) and (ii) the behavioral data indicated that intrapersonal emotion regulation occurred on the relevant trials confirms that the present paradigm provided a valid intrapersonal emotion regulation task. The necessity of setting up the paradigm in this manner was driven by the aim of directly comparing intra- and interpersonal scans. Although not having the conspecific present in the corner of the screen during the intrapersonal scan would undoubtedly been more consistent with previous paradigms investigating intrapersonal ER, this would have prevented any direct comparison with the interpersonal run (e.g., any differences found could simply have been a function of the inclusion of another person on the screen). Future studies could further investigate potential differences by using paradigms that manipulate whether participants' attempts to regulate their own emotion occur in the presence of other individuals or not. This is particularly interesting question given that the majority of “real world” emotion regulation is performed in front of an actual or imagined audience (Erber et al., 1996).

We also acknowledge that due to the exploratory nature of this study, we have made use of “reverse inference” in the interpretation of some of our findings. Such inferences have been said to be limited when the selectivity of the regions discussed cannot be established (Poldrack, 2006). However recent work suggests that emotion processing recruits a range of mental processes that are also used for other purposes, such as empathy and mentalizing (e.g., Lindquist et al., 2012). Within such a framework it is the combination of these processes that differentiates the different types of emotion regulation and distinguishes it from other processes. Future studies into interpersonal emotion regulation that systematically manipulates the proposed component factors could help provide a more in-depth exploration.

### Implications

The results of the present study may help to understand conditions in which both intra- and interpersonal emotion regulatory processes are dysfunctional, such as schizophrenia. Our findings suggest that interpersonal emotion regulation shares overlapping neural substrates with intrapersonal emotion regulation, but also involves activation of areas previously implicated in affective and cognitive components of empathy. This may help to explain why deficits in these processes, such as those that have previously been observed in disorders such as schizophrenia (e.g., Lee et al., 2004, 2006), may partially manifest as a reduced capacity for interpersonal emotion regulation and hence problems with social interaction. Such an interpretation fits with the idea that schizophrenia can be characterized as a dysfunction of interpersonal functioning (Frith, 1992). Further studies using similar paradigms with clinical groups would help to understand how specific deficits in interpersonal emotion regulation relate to more general deficits in interpersonal interactions.

## Conclusion

The present research finds an overlapping pattern of activation with an interpersonal emotion regulation task and an equivalent intrapersonal emotion regulation task, incorporating a fronto-parietal network. However, as well as the similarities involved in both processes, there were additional activations associated with interpersonal emotion regulation in brain areas that have previously been shown to be involved in the affective and cognitive components of empathy. This was particularly the case when the strategy used to influence the emotional state of the other person was cognitive reappraisal rather than expressive suppression.

### Conflict of interest statement

The authors declare that the research was conducted in the absence of any commercial or financial relationships that could be construed as a potential conflict of interest.

## Footnotes

^1^During both scans the video-link of the other person watching the films was pre-recorded, although participants were informed that it was a live video-link.

^2^Although vocalization was only strictly necessary for the interpersonal task (i.e., in order to meet the requirements of an interpersonal emotion regulation task; see Zaki and Williams, 2013), participants were also instructed to vocalize their chosen strategy for emotion regulation in the intrapersonal condition in order to match motor movements related to speaking across the conditions.

^3^Analysis of the behavioral data revealed no differences between ‘successful’ and ‘unsuccessful’ regulation trials in the perceived affect of the other person. This lack of difference, in addition to the relatively small number of ‘unsuccessful’ trials, meant that we did not model these events separately in the analysis of the fMRI data.
